# Cost-effectiveness analysis of additional local prostate radio therapy in metastatic prostate cancer from a medicare perspective

**DOI:** 10.1186/s13014-024-02544-0

**Published:** 2024-11-21

**Authors:** Kristina K. M. Kramer, Nina-Sophie Schmidt-Hegemann, Thilo Westhofen, Marco Foglar, Jens Ricke, C. Benedikt Westphalen, Marcus Unterrainer, Wolfgang G. Kunz, Dirk Mehrens

**Affiliations:** 1 Department of Radiology, LMU University Hospital, LMU Munich, Munich, Germany; 2 Department of Radiation Oncology, University Hospital, LMU Munich, Munich, Germany; 3 Department of Urology, Ludwig-Maximilians-University of Munich, Munich, Germany; 4 Laser-Forschungslabor, LIFE Center, University Hospital, LMU Munich, Munich, Germany; 5 Comprehensive Cancer Center and Department of Medicine III, Ludwig Maximilian University of Munich, Munich, Germany; 6 Department of Nuclear Medicine, LMU University Hospital, LMU Munich, Munich, Germany

**Keywords:** Cost-effectiveness, Prostate cancer, Radiotherapy, Bone metastases, NRLN metastases

## Abstract

**Background:**

Metastatic prostate cancer remains a therapeutic challenge. Based on data of the STAMPEDE trial, patients with a low metastatic burden showed prolonged failure-free and overall survival when treated with prostate radio therapy (RT) in addition to standard of care (SOC). The objective of this study was to determine the cost-effectiveness of additional prostate RT compared to SOC alone for following subgroups: non-regional lymph node (NRLN) metastases, up to three bone metastases and four or more bone metastases.

**Methods:**

A partitioned survival model was implemented with clinical data from STAMPEDE trial. Analyses were performed from a United States healthcare system perspective. Costs for treatment and adverse events were derived from Medicare coverage. Utilities for health states were derived from public databases and literature. Outcome measurements included incremental costs, effectiveness, and cost-effectiveness ratio. The willingness-to-pay threshold was set to USD 100,000 per quality-adjusted life year (QALY).

**Results:**

Additional RT led to 0.92 incremental QALYs with increased costs of USD 26,098 with an incremental cost-effectiveness ratio (ICER) of USD 28,452/QALY for patients with only NRLN metastases and 3.83 incremental QALYs with increased costs of USD 153,490 with an ICER of USD 40,032/QALY for patients with up to three bone metastases. Sensitivity analysis showed robustness of the model regarding various parameters. In probabilistic sensitivity analysis using Monte Carlo simulation with 10,000 iterations, additional RT was found as the cost-effective strategy in over 96% for both subgroups iterations at a willingness-to-pay threshold of USD 100,000/QALYs.

**Conclusions:**

Additional RT is cost-effective in patients with only NRLN metastases and up to three metastases compared to SOC.

**Supplementary Information:**

The online version contains supplementary material available at 10.1186/s13014-024-02544-0.

## Background

Prostate cancer is the most common malignant disease in men with an average incidence rate of 115/100,000 in the United States presenting the second most common cause of death by cancer with 299,010 new cases and 35,250 fatalities expected for 2024 [1].

Despite of the excellent survival rate of localized tumors with a 5-year survival of 97% [1], men with metastatic tumor are only expected for a 30.7% 5-year survival-rate [2]. With 7.2% of men being diagnosed in a metastatic state [3], adequate therapy strategies are essential.

Numerous retrospective analyses concluded a positive association between survival and the additional use of radiotherapy (RT) of the primary tumor in men with metastatic prostate cancer [4–7]. The randomized STAMPEDE (Systemic Therapy in Advancing or Metastatic Prostate Cancer: Evaluation of Drug Efficacy) trial was conducted concluding a survival advantage for patients with low metastatic burden without suggesting a threshold for metastases [8]. In a subsequent subgroup analysis by Ali et al. standard of care with additional RT (SOC + RT) has proven to improve survival with low-burden metastatic prostate cancer patients [9].

Despite efficacy, cost is a matter of essential importance. In these studies, no information on the economic perspective was assessed. Annually, health costs attributable to metastatic prostate cancer amounted to between USD 5.2 and USD 8.2 billion for the years 2007 until 2017 in the US [10]. This study thrives to analyze the cost-effectiveness of systemic therapy in combination with radiotherapy compared to sole systemic therapy for Medicare-eligible metastatic prostate cancer patients in the United States (US) based on results of the STAMPEDE trial.

## Materials and methods

### Data pool and patient characteristics

#### Data pool

Clinical information was derived from the randomized phase III clinical STAMPEDE trial [8] as well as from secondary analysis by Ali et al.[9]. Standard of Care (SOC) was life-long androgen deprivation therapy (ADT) with luteinizing hormone releasing hormone analogues in 99% of patients, 18% received additionally docetaxel [8]. After progress, the medication was altered [8]. Additional RT was administered either with 55 Gy in 20 daily fractions over four weeks or 36 Gy in 6 weekly fractions over 6 weeks [8, 9].

The secondary analysis by Ali et al. revealed a benefit for patients with up to three bone metastases and only NRLN metastases on overall survival (OS) and failure-free survival (FFS) when treated with additional RT, for all other subgroups (including ≥ 4 bone metastases) no significant benefit was revealed [9].

#### Patient characteristics

2061 patients with newly diagnosed metastatic prostate cancer were randomized in ratio 1:1 into two therapy arms. After exclusion, 976 patients treated with SOC were compared to 963 patients treated with additional radiotherapy (SOC + RT) [8, 9].

The median age at diagnosis was 68 years, ranging from 63 to 73 years [8, 9]. Within the 1939 included patients, 82% featured bone metastases with or without additional non-regional lymphatic node (NRLN) metastasis. 9% of the patients only showed NRLN metastasis (M1a), 9% presented with other metastases (e.g. visceral) [9].

### Model structure

**Fig. 1:**
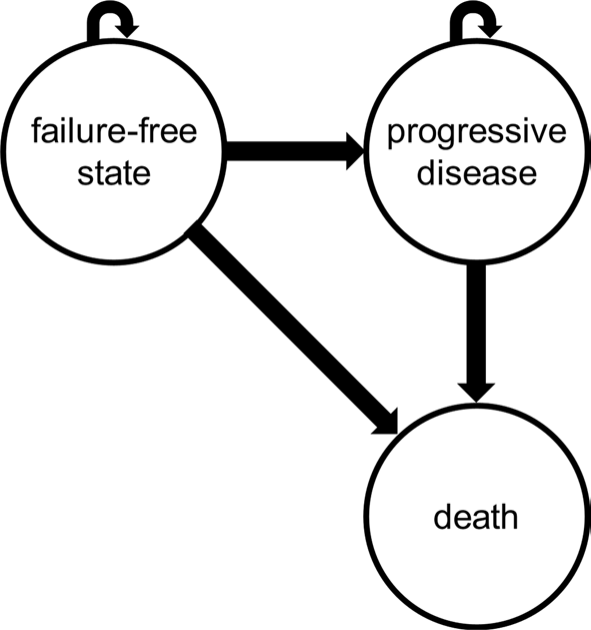
State-transition diagram. State-transition diagram for each of the groups over time intervals. Patients in a failure-free state can either stay in failure-free state, experience progression (progressive disease), or die (death). After progression, patients can either remain progressive or die. Death is the absorbing state

A partitioned survival analysis (PSA) model was developed and implemented using a decision-analytic software (TreeAge Pro Healthcare Version 2021, TreeAge Software, LLC.) [11]. Patients were distributed to sub-cohorts according to their metastatic burden of only NRLN, ≤ 3 bone metastases and ≥ 4 bone metastases. Cycle length was set to one month. At the beginning of the simulation all patients started in failure-free state. During each cycle, patients could either remain in their failure-free state (FFS), transit to a post-progression state (PPS) or die. Death is the terminal state (see Fig. 1). The analysis ceased, when over 99% of the subpopulations reached death state. For an illustration of the implemented PSA see supplementary Table S1. The analysis was carried out according to the CHEERS guidelines [12].

### Model input parameters

#### Progression and survival probabilities

Kaplan–Meier survival data were sourced from Ali et al. [9] as graphical data and extracted using Engauge Digitizer Software (Engauge Digitizer, Version 12.1, Mark Mitchell et al., Torrance California, United States, Open Source) [13]. A conversion of the resulting data to a patient-wise format and the fitting of parametric distributions [14] to the survival values was carried out with statistical programming language R (R, version 4.2.1, R Core Team, Vienna, Austria, Open Source) [15]. The best coinciding distributions to the Kaplan–Meier curves were visually controlled. For detailed information about the resulted parameters for the distributions see Table 1. Thus, there are no absolute transition probabilities but time-dependent transitional probabilities according to the extracted distributions from the Kaplan–Meier data.

**Table 1:** Detailed input parameters

Model Input	Value(s) [95% CI]	Distribution	Reference/Details
***Initial Probabilities***			
Failure-free state	1		
Progressive disease	0		
Death	0		
***Survival distributions***			
**SOC**			[9, 13–15]
FFS only NRLN metastases	mean = 3.047 [2.784; 3.311] sd = 1.267 [1.062; 1.510]	LogNormal	
OS only NRLN metastases	shape = 0.068548 [0.042152; 0.094944] rate = 0.001768 [0.000726; 0.004308]	Gompertz	
FFS ≤ 3 metastases	shape = 0.00417 [-0.00111; 0.00945] rate = 0.02929 [0.02596; 0.03305]	Gompertz	
OS ≤ 3 metastases	shape = 0.043778 [0.036064; 0.051491] rate = 0.003057 [0.002378; 0.003931]	Gompertz	
FFS ≥ 4 metastases	mean = 2.3222 [2.2513; 2.3932] sd = 1.1322 [1.0794; 1.1877]	LogNormal	
OS ≥ 4 metastases	mean = 3.7020 [3.6392; 3.7648] sd = 0.8840 [0.8299; 0.9417]	LogNormal	
**SOC** + **RT**			[9, 13–15]
FFS only NRLN metastases	mean = 3.575 [3.323; 3.828] sd = 1.135 [0.931; 1.383]	LogNormal	
OS only NRLN metastases	shape = 0.070110 [0.040431; 0.099789] rate = 0.001177 [0.000417; 0.003323]	Gompertz	
FFS ≤ 3 metastases	mean = 3.6178 [3.5269; 3.7087] sd = 1.2795 [1.2010; 1.3630]	LogNormal	
OS ≤ 3 metastases	mean = 4.3119 [4.2266; 4.3972] sd = 0.7626 [0.6934; 0.8386]	LogNormal	
FFS ≥ 4 metastases	mean = 2.4377 [2.3659; 2.5095] sd = 1.1448 [1.0911; 1.2011]	LogNormal	
OS ≥ 4 metastases	mean = 3.6211 [3.5672; 3.6751] sd = 0.7869 [0.7412; 0.8353]	LogNormal	
***Health Care Costs***			
**SOC**			[8, 16, 17]
Initial average cost	USD 3,279 (Additional therapy)		
Monthly average cost	USD 3,040 (LHRH agonists, dual androgen blockage)		
**SOC Post Progression**			[8, 16, 17]
Initial average cost	USD 9,619 (Additional therapy)		
Monthly average cost	USD 3,069 (LHRH agonists, additional therapy)		
**RT 20 fractions**			[16, 19, 20]
Total medicare fee	USD 12,128		
**RT 6 fractions**			[16, 19, 20]
Total medicare fee	USD 7,430		
***Utilities***			
SOC	0.82		[22–24]
SOC + RT	0.90		[25]
Late toxicities	0.99*		[21, 23, 25, 26]
Progress	0.6325		[21, 23–25]

*Adjusted for 1.72% of patients experiencing late genitourinary and 2.43% of patients experiencing late gastrointestinal toxicities, see supplementary Table S2

Survival curves were derived from the secondary analysis of Ali et al. [9] of STAMPEDE trial [8]. Utilities and disutilities were derived from literature. Costs were derived from medical pricing e.g. Medicare. Ranges for deterministic sensitivity analysis were determined by the 95% confidence interval of the initial probabilities and by ± 20% for costs. All costs were converted to 2022 USD. sd = standard deviation, CI = confidence interval.

#### Costs

The analysis was performed from a United States healthcare system perspective. Standard treatment costs for failure-free state and post-progression state were derived from Medicare and Medicaid Spending by Drug datasets (65 years or older) between 2016 and 2020 [16] in combination with pricing information from the Centers for Medicare & Medicaid Services [17]. Costs for 2020 were chosen, all costs were adjusted to the consumer price index of the United States for 2022. A discount rate of 3% annually was added for this investigation. Costs are summarized in Table 1, for detailed version see in supplementary Table S1. Obtained values were plausibility-checked with Wang et al. (2022) [18].

##### Standard of care cost

Costs for standard of care treatment for failure-free state and post-progression state were calculated referring to the drug administration in the STAMPEDE trial in consultation of the Department of Urology of the University Hospital, LMU Munich [8].

Standard of care in the failure-free state was luteinizing hormone releasing hormone (LHRH) agonists for all participants [8]. Additionally, 18% of patients were treated with docetaxel, remaining 82% of patients were assumed to have either received treatment with enzalutamide, apalutamide or abiraterone. Each possible treatment cost was multiplied by the percentage of the patients having received that treatment, resulting in average initial cost (added once) and average recurring cost (added monthly).

Medication cost after progressive disease (treatment failure) was calculated as stated in the STAMPEDE trial (supplementary Table S5) [8]: Standard of care treatment in post-progression state was LHRH agonists for all participants. Additionally, 21% of patients received abiraterone, 6% received cabazitaxel, and 33% received docetaxel. Remaining patients (60%) were assumed to have enzalutamide treatment. Total costs were specified as initial costs and monthly costs.

##### Radio therapy cost

Cost calculation was based on the NCCN guidelines for low volume M1 disease [19] for external beam radiation therapy (EBRT, more precisely intensity-modulated radiation therapy, IMRT) treatment according to RADIATION ONCOLOGY CPT® [20], HCPCS CODES BY PROCESS OF CARE “[20] and CMS.gov (The Centers for Medicare & Medicaid Services) [16] in consultation with the Department of Radiation Oncology of the University Hospital, LMU Munich. Total costs for the RT treatment were calculated for both eligible RT schemes. The price for a 20 fractions RT amounted to USD 12,128, the second schedule with a 6 fractions RT amounted to USD 7,430. For our analysis the more costly RT scheme with 20 fractions was applied with an additional base case analysis for the RT scheme with 6 fractions.

Therapy-related late toxicity of grade 3 and 4 was seen in 41 cases of patients who received RT (n = 988) [8]. Disutilities (converted to utilities) were added as initial costs at the beginning of the therapy.

#### Utilities and disutilities

Utility values for FFS and PPS were derived from literature [21–25] and calculated as average values for the failure-free state and the post-progression state. Values for toxicity were collected from literature [21, 23, 25, 26], average values were calculated and weighted according to the reported late radiotherapy toxicity scores in Parker et al. [8]. Utility weights can be found in Table 1, the composition of the utility values can be found in supplementary Table S2. A discount rate of 3% annually was set for the utilities.

### Cost-effectiveness analysis

SOC and SOC + RT were compared according to costs, their effectiveness (quality-adjusted life years, QALYs), incremental cost-effectiveness ratio (ICER) and net monetary benefit (NMB). Incremental costs, utilities and ICERs were calculated for each patient subgroup and their received therapy separately. The willingness-to-pay threshold was set to 100.000 USD per QALY. This threshold value classifies medical services in terms of reimbursability [27]. For more details see supplementary text.

### Sensitivity analyses

To check the robustness of the model, comprehensive deterministic and probabilistic sensitivity analyses were executed for the more expensive radiotherapy regime (20 fractions RT). Deterministic sensitivity analysis with ranges for initial probabilities by the 95% confidence interval and costs by ± 20%. The probabilistic sensitivity analysis allowed alterations of multiple input parameters using Monte Carlo simulation runs (n = 10,000). Model input parameters were assigned the appropriate distributions, see Table 1. Treatment costs were modeled using gamma distribution. Beta distribution was used for utility values, PFS and OS data.

## Results

### Base case analysis

For the populations with NRLN metastases and up to three bone metastases, additional RT led to an increase of QALYs, accompanied by augmented costs. Detailed information can be found in Table 2.

**Table 2:** Base case analysis

	Treatment arm	Cost (USD)	QALYs	Increased cost (USD)	Increased QALYs	ICER (USD/QALY)	NMB (USD)
***SOC vs. SOC*** + ***RT (20 fractions RT)***							
Only NRLN metastases	SOC	154,788	2.89				-10,312
	SOC + RT (20 fractions)	180,886	3.81	26,098	0.92	28,452	9,453
≤ 3 bone metastases	SOC	173,899	3.24				-12,078
	SOC + RT (20 fractions)	327,389	7.07	153,490	3.83	40,032	26,144
≥ 4 bone metastases	SOC	195,501	3.44	14,862	0.03	478,797	-23,539
	SOC + RT (20 fractions)	180,639	3.41				-10,230
***SOC vs. SOC*** + ***RT (6 fractions RT)***							
Only NRLN metastases	SOC	154,788	2.89				-10,312
	SOC + RT (6 fractions)	176,188	3.81	21,400	0.92	23,261	14,312
≤ 3 bone metastases	SOC	173,899	3.24				-12,078
	SOC + RT (6 fractions)	322,691	7.07	148,792	3.83	38,849	30,809
≥ 4 bone metastases	SOC	195,501	3.44	19,559	0.03	615,967	-23,539
	SOC + RT (6 fractions)	175,941	3.41				-10,230

Results of the cost-effectiveness analysis. For only NRLN metastases SOC + RT was associated with an ICER of USD 28,452 (20 fractions RT) and USD 23,261 (6 fractions RT). For ≤ 3 bone metastases SOC + RT was associated with an ICER of USD 40,032 (20 fractions RT) and USD 38,849 (6 fractions RT). WTP was set to USD 100,000/QALY. The difference in price of the costs of RT with 20 fractions and 6 fractions equals to USD 4,698 for each subgroup.

Concerning the subpopulation with NRLN metastases, effectiveness increased by 0.92 QALYs. For the 20 fractions RT, increased costs were USD 26,098, the corresponding ICER amounted to USD 28,452 per QALY and the net monetary benefit (NMB) was USD 9,453. With the 6 fractions RT, additional costs were USD 21,400, the ICER was USD 23,261 and the NMB was USD 14,312.

Within the subpopulation of three or less bone metastases the QALY increased by 3.83. For the 20 fractions RT, increased costs were USD 153,490. The base-case analysis showed that the corresponding ICER per QALY equaled to USD 40,031, the NMB amounted to USD 26,144. With the 6 fractions RT, additional costs were USD 148,792, the ICER was USD 38,849 and the NMB was USD 30,809.

Concerning the subpopulation of 4 or more bone metastases, no cost-effectiveness was found for both RT schedule.

### Deterministic sensitivity analysis

**Fig. 2:**
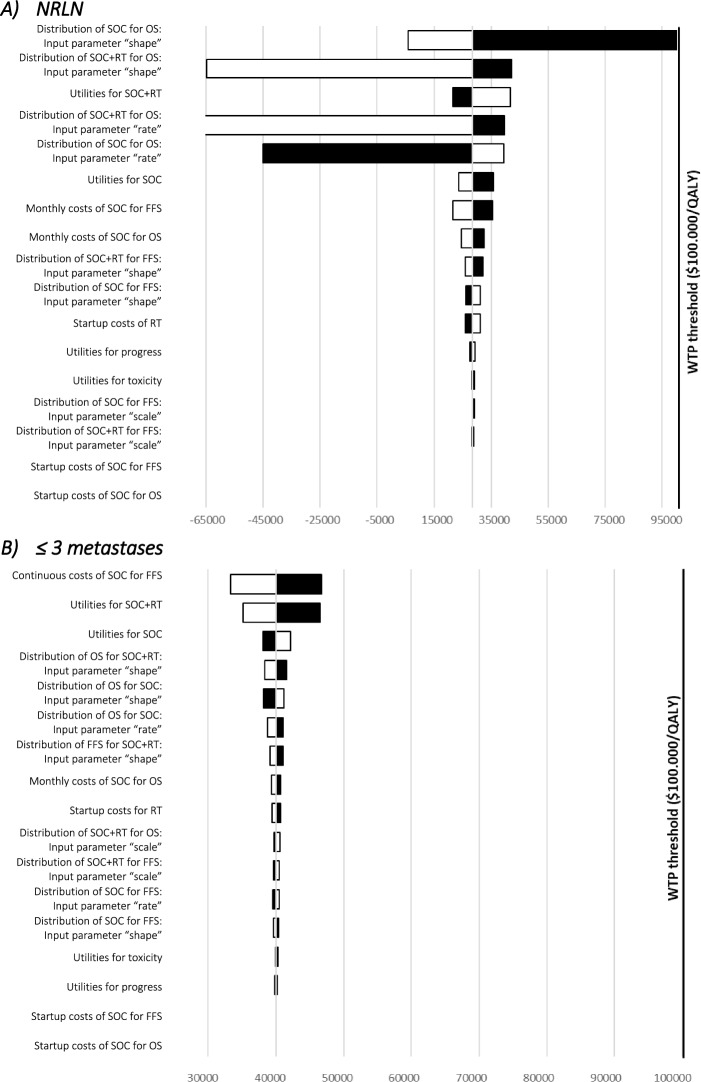
Tornado diagrams for SOC and SOC + RT (20 fractions). Tornado diagrams for deterministic sensitivity analyses. Tornado diagrams in the one-way sensitivity analysis depict the effect of variation of only one model parameter per simulation on the model outcome. The impact of the parameters on the outcome is sorted in descending order. Parameters for survival distributions showed the strongest cumulative impact on the ICER for the subgroup of only NRLN metastases. For the subgroup of ≤ 3 metastases continuous costs of SOC for failure-free survival possessed the strongest impact on the ICER followed by the utilities for SOC + RT (20 fractions) and SOC. Black bars indicate changes based on the upper bound of a parameter variation, and white bars indicate the lower bound of the respective parameter. WTP thresholds of USD 100,000/QALY are depicted

The results of the deterministic one-way sensitivity analyses are presented in Fig. 2. In only NRLN metastatic patients (A), parameters for survival distributions for SOC and SOC + RT demonstrated the strongest impact on the ICER. For the subgroup of up to three bone metastases (B), changes in parameters in general only had a minor impact on the ICER compared to the subgroup of only NRLN metastases. Continuous costs for SOC during failure-free survival influenced the ICER the most, followed by utility values for SOC + RT and SOC. Variations of RT costs (added as startup costs) showed only a minor influence on the ICER for both presented patient cohorts (Fig. 2).

### Probabilistic sensitivity analysis

**Fig. 3:**
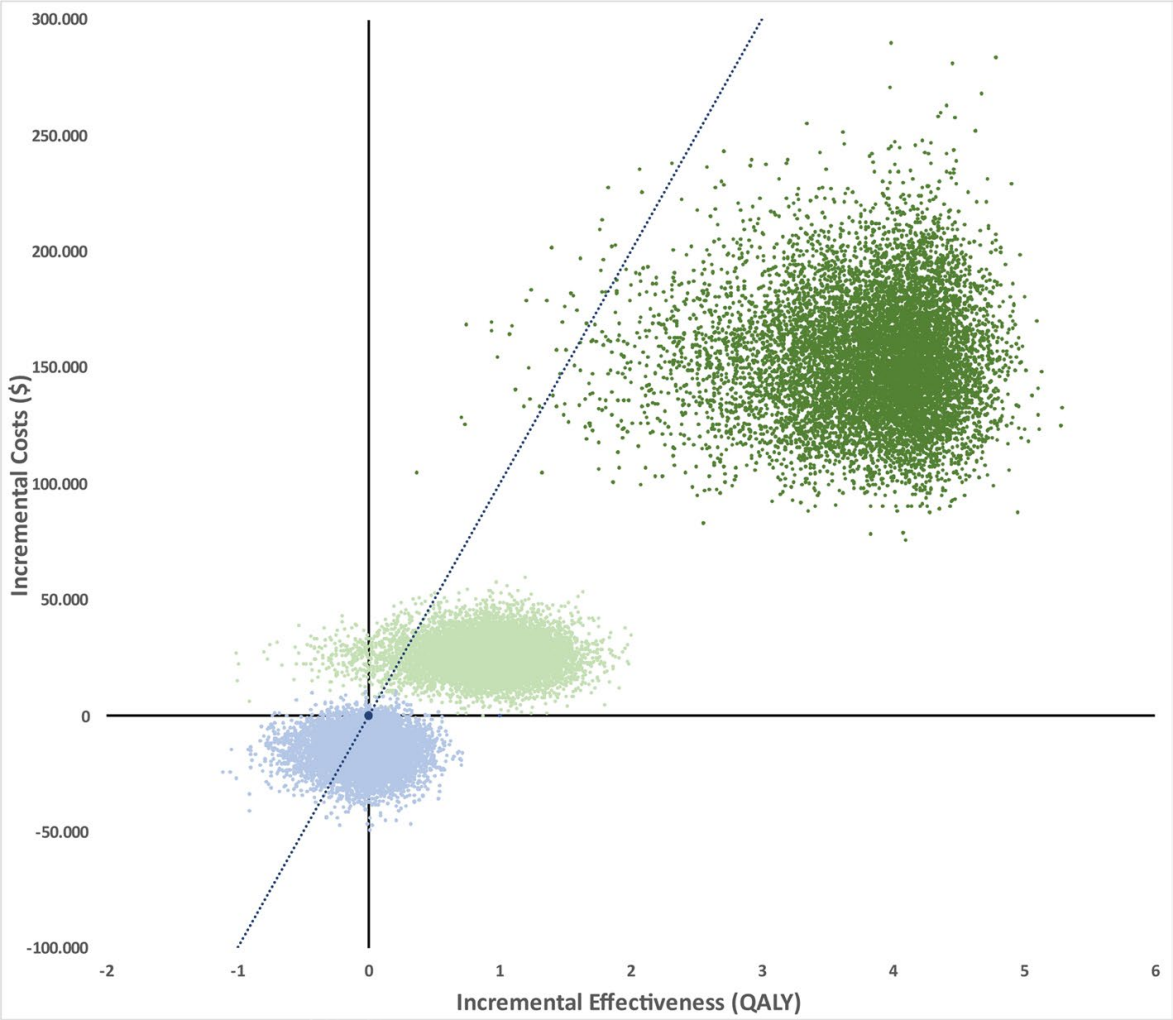
Monte Carlo simulations – Graphical results for SOC vs. SOC + RT (20 fractions). Probabilistic sensitivity analysis. Cost-effectiveness planes of incremental costs and incremental effectiveness of SOC vs. SOC + RT (20 fractions) for the probabilistic sensitivity analysis. Each dot represents one simulation run. The dashed line indicates a WTP threshold of 100,000 USD/QALY. Dots to the right of this line are considered cost-effective simulation runs. Simulations for NRLN are colored in mint-green, ≤ 3 bone metastases are colored in dark-green, ≥ 4 bone metastases are colored in light-blue

The results of the probabilistic sensitivity analysis are presented in Fig. 3. At a NRLN state, 86% of simulations showed that SOC + RT was the preferred strategy, resulting in better outcomes with minimally higher costs. Furthermore, the mean values for the ICER were in 96.72% < 100,000 USD/QALY. At a state with three or less bone metastases, in 85% of simulations SOC + RT was the preferred strategy. The mean values for the ICER were in 99.61% below the willingness-to-pay threshold, see supplementary Table S4.

## Discussion

### General

This study evaluated the economic impact regarding the cost of radiotherapy in addition to systemic therapy for newly diagnosed oligometastatic prostate cancer patients, indicating that SOC + RT is a cost-effective treatment option compared to SOC alone for two of the three analyzed subgroups (NRLN metastases, ≤ 3 metastases). For all other subgroups, no benefit was found. The analyses showed robust results with most of Monte Carlo simulations below the willingness-to-pay threshold. With longer failure-free survival, additional costs of RT were partly amortized due to lower treatment costs for failure-free state.

### Subgroup discussion

#### ≤ 3 metastases

SOC + RT was most effective for patients with three or less bone metastases, amounting to higher incremental benefits and a positive NMB. The patients benefited from approximately four years of accumulated perfect health gained. Although costs of SOC + RT accounted to almost double of SOC costs, mean values for the ICER were below the willingness-to-pay threshold in over 99% of simulations. All in all, SOC + RT is the preferred strategy at this state.

#### Only NRLN metastases

SOC + RT was effective for patients with only NRLN metastases, amounting to higher incremental benefits and a positive NMB. The patients benefited from approximately one year of accumulated perfect health gained for 17% higher costs of SOC + RT compared to SOC alone. The mean values for the ICERs were almost entirely below 100,000 USD/QALY. Therefore, additional RT was cost-effective at the NRLN metastatic state. SOC + RT is the preferred strategy at this state. Compared to patients with up to three bone metastases, the benefit of RT in this subgroup was slightly reduced.

#### ≥ 4 metastases

For the subpopulation with four or more bone metastases, no cost-effectiveness of SOC + RT was found. In this subgroup, most cases profited less at lower costs when adding RT to SOC. For both therapy strategies SOC and SOC + RT, the net monetary benefit was negative. For SOC + RT, QALYs decreased by 0.03 compared to SOC alone. SOC + RT was 8.2% less costly than SOC alone. For patients receiving SOC + RT, Ali et al. found a hazard ratio of 1.08 for experiencing death [9]. As patients have a higher risk of dying earlier when receiving additional RT, payment for more expensive post-progression therapies is lower, resulting in lower overall costs. Notably, Ali et al. [9] did not find significant results for the hazard ratio for OS for the subgroup of four or more bone metastases, resulting in a need for further investigation. In conclusion, a difference of 0.03 QALYs does not show a clinically relevant advantage.

### Deterministic sensitivity analysis

The reason for strong impact of variation of distributional input parameters and low impact of variation of costs can be explained likewise. A variation by the 95% confidential interval for the distribution changes the distribution significantly resulting in differing survival estimations. On the other hand, a 20% increase or decrease in costs does not affect the calculation as much as a vastly deviating survival rate.

### External validation

#### Comparison of study design

The STAMPEDE trial explicitly stated that additional prostate RT improved OS only in certain subgroups [8]. Lester-Coll et al. [25] evaluated the cost effectiveness of RT + SOC based on the original STAMPEDE trial [8] without subgroup analyses. They stated that the longer patients survived the more cost-effective the treatment was, because patients with advanced disease stages already died and, thus, only patients with limited disease seemed to have profited [25]. In our study, the subgroup analyses provided more detailed and more applicable results. Further differences to our study included the usage of a Markov Model and permission to apply cost for progression twice [25].

#### Comparison of costs

Comparing our input parameters for costs to other publications, our monthly costs for failure-free survival matched cost estimations of Wang et al. [18]. Lester-Coll et al. [25] estimated the cost for SOC at USD 63, which does not seem to reflect true costs for SOC and might be based on alternative therapy regimes. For progressive disease, our costs considerably deviated to Wang et al. [18], where higher monthly costs were seen. Since costs for progressive disease do not differ between SOC and SOC + RT, the impact of this difference does not need to affect the outcome per se.

For external validation, we compared our base case results to Clarke et al. [28] where costs for metastatic disease varied from USD 62,802 to USD 157,856, compared to our average value of USD 174,729. Costs were applied to SOC only and SOC combined with abiraterone and prednisolone, both permitted therapy schemes in the STAMPEDE trial.

Generally, the model showed good robustness concerning costs, reassuring the external validity of our results. Furthermore, our costs were calculated rather than quoted, as practiced in other publications. Detailed information concerning our input parameters can be found in supplementary Table S5.

#### Comparison of base case analysis

When comparing our base case analysis with results from Lester-Coll et al. [25], costs and QALYs differ. In Lester-Coll et al. [25], additional RT does come with cheaper overall costs, mainly due to high costs combined with disease progression, see supplementary Table S5. This might be caused by the difference in progressive disease states (one state in our study vs. two states in Lester-Coll et al. [25]) and costs for progression. Having in mind the limitations of lacking subgroup analysis, lifetime QALYs of both studies are comparable (0.81 QALYs in Lester-Coll et al. vs. 0.03–3.83 QALYs in this study). While Lester-Coll et al. [25] showed a smaller advantage for all groups, our study could identify groups with the strongest benefit. For brief overview see Table 3, for detailed overview see supplementary Table S6. Generally, both studies showed a benefit for additional RT, affirming the usefulness of additional radiation treatment to SOC alone.

**Table 3:** Comparison of costs (extended view see supplementary Table S4)

	Cost type	Kramer et al. [USD]	Wang et al. [18] [USD]	Lester-Coll et al. [25] [USD]	Clarke et al. [28] [USD]
***SOC***					
mCSPC	One-time cost	3,279			
	Monthly cost	3,040	3,647	63	
mCRPC (≈ progressive disease)	One-time cost	9,619			
	Monthly cost	3,069	12,296		
mCSPC	Base case results	154,788 173,899 195,501 average: 174,729		328,971	157,856 62,802
***RT***					
20 fractions	One-time cost	12,128		16,338	
6 fractions	One-time cost	7,430		9,659	
***SOC*** + ***RT***					
mCSPC	Base case results	180,886 327,390 180,639 average: 229,638		298,741	

Comparison of costs. A short overview of assumed costs from different studies. Costs are shown in USD.

In conclusion, base case results for SOC differ between different studies (SOC: USD [62,802;328,971] [25, 28]). Our findings rank in the middle amongst these values.

#### Comparison of utilities

When comparing utilities, it is to be noted that multiple sources were used to calculate an averaged value used in our analysis, while other publications rather used individual values derived from other studies. For details see supplementary Table S2 and S5.

### Limitations

Preparations of survival data required extensive pre-processing which included fitting survival curves to parametric distributions which might cause variation between the original data and the used input data. Addressing this, a comparison of the fitted parametric curves to the extracted survival values and a visual comparison was performed, see supplementary Figure S2.

As we derived FFS and OS directly from Kaplan Meier data, a Markov model would have required further assumptions about the post-progression survival. In literature, the results of PSA models and Markov models are seen as equally valid approaches [29]. Pursuing our goal of an accurate model we deliberately opted for the PSA approach.

Ali et al. does not contain information about absolute number or volume of lymphatic or visceral metastases [9], preventing a closer analysis in these subgroups.

Our results are based on Medicare pricing which only patients with an age of 65 or above are eligible for [30]. Patients in the STAMPEDE cohort featured an age in the interquartile range from 63 to 73 [8, 9], indicating that not all patients were eligible for this specific health insurance. Nevertheless, this practice is well used in other publications [18, 25].

Our analysis was performed in the most cautious way, tending to overestimate therapy cost for RT by using the more expensive RT scheme with 20 fractions as base of discussion. Still, the ICER for the 20 fractions RT schedule were in over 95% of probabilistic sensitivity analysis below the threshold of USD 100,000/QALY. Furthermore, the more affordable SOC + RT treatment arm with the 6 fractions RT schedule was as cost-effective as the more expensive schedule with cost reductions of USD 4,698 in the base case analysis. Thus, this study slightly underestimates the cost-effectiveness of additional local prostate RT in metastatic prostate cancer. Deterministic sensitivity analysis demonstrated only a minor effect of RT costs on our analysis.

### Clinical context of additional RT in patients with metastatic prostate *cancer*

Further studies besides STAMPEDE confirm a positive effect of additional prostate RT in men with metastatic prostate cancer. Furthermore, Ali et al. [31] recommended prostate radiotherapy as first-line treatment option in de novo low metastatic patients. In this context, our study revealed patient subgroups in which additional RT not just improves survival but is also cost-effective, providing reliable data for future patient selection.

## Conclusion

According to our study, additional administration of prostate RT to SOC treatment was seen as a cost-effective treatment for patients with NRLN metastases and for patients with up to three bone metastases. These results might guide clinical decision making for certain subgroups of prostate cancer patients, selecting the most effective treatment for each patient, saving resources, and maximizing the benefits for the entire health care sector.

## Supplementary Information

Additional file1.

## Data Availability

No datasets were generated or analysed during the current study.

## Abbreviations

ADT: Androgen deprivation therapy
CI: Confidence interval
FFS: Failure-free survival
EBRT: For external beam radiation therapy
ICER: Incremental cost-effectiveness ratio
IMRT: Intensity-modulated radiation therapy
LHRH: Luteinizing hormone releasing hormone
NMB: Net monetary benefit
NRLN: Non-regional lymph node
OS: Overall survival
PSA: Partitioned survival analysis
PPS: Post-progression state
RT: Prostate radio therapy
QALYs: Quality-adjusted life years
sd: Standard deviation
SOC: Standard of care
SOC + RT: Standard of care with additional RT
STAMPEDE: Systemic Therapy in Advancing or Metastatic Prostate Cancer: Evaluation of Drug Efficacy
USD: United States Dollar
