# Supplementary material for: Cost-effectiveness analysis of additional local prostate radio therapy in metastatic prostate cancer from a medicare perspective
Source: Radiat Oncol. 2024 Nov 21;19:167. doi: 10.1186/s13014-024-02544-0 (PMC11580356; doi:10.1186/s13014-024-02544-0)
Supplement: Supplementary file 1 — Additional file1. [file 13014_2024_2544_MOESM1_ESM.docx]

Supplement

[Supplementary figure S1 | PSA-Model 2](#_Toc158220513)

[Supplementary table S1 | Detailed cost calculation 3](#_Toc158220514)

[SOC FFS [1, 2] 3](#_Toc158220515)

[SOC post-progression [1, 2] 3](#_Toc158220516)

[RT 20 fractions [1, 3, 4] 3](#_Toc158220517)

[RT 6 fractions [1, 3, 4] 3](#_Toc158220518)

[Supplementary table S2 | Detailed utility calculations 4](#_Toc158220519)

[Supplementary table S4 | Monte Carlo simulations – Numeric results 4](#_Toc158220520)

[Supplementary table S5 | Comparison with other CEAs 5](#_Toc158220521)

[Supplementary table S6 | Comparison of base case analysis with Lester-Coll [18] 6](#_Toc158220522)

[Supplementary figure S2 | Parametric distributions 7](#_Toc158220523)

[Supplementary text 10](#_Toc158220524)

[Introduction 10](#_Toc158220525)

[Therapies 10](#_Toc158220526)

[Materials and Methods 10](#_Toc158220527)

[Cost-Effectiveness Analysis 10](#_Toc158220528)

[Discussion 10](#_Toc158220529)

[Clinical context of additional RT in patients with metastatic prostate cancer 10](#_Toc158220530)

[References 11](#_Toc158220531)

# Supplementary figure S1 | PSA-Model


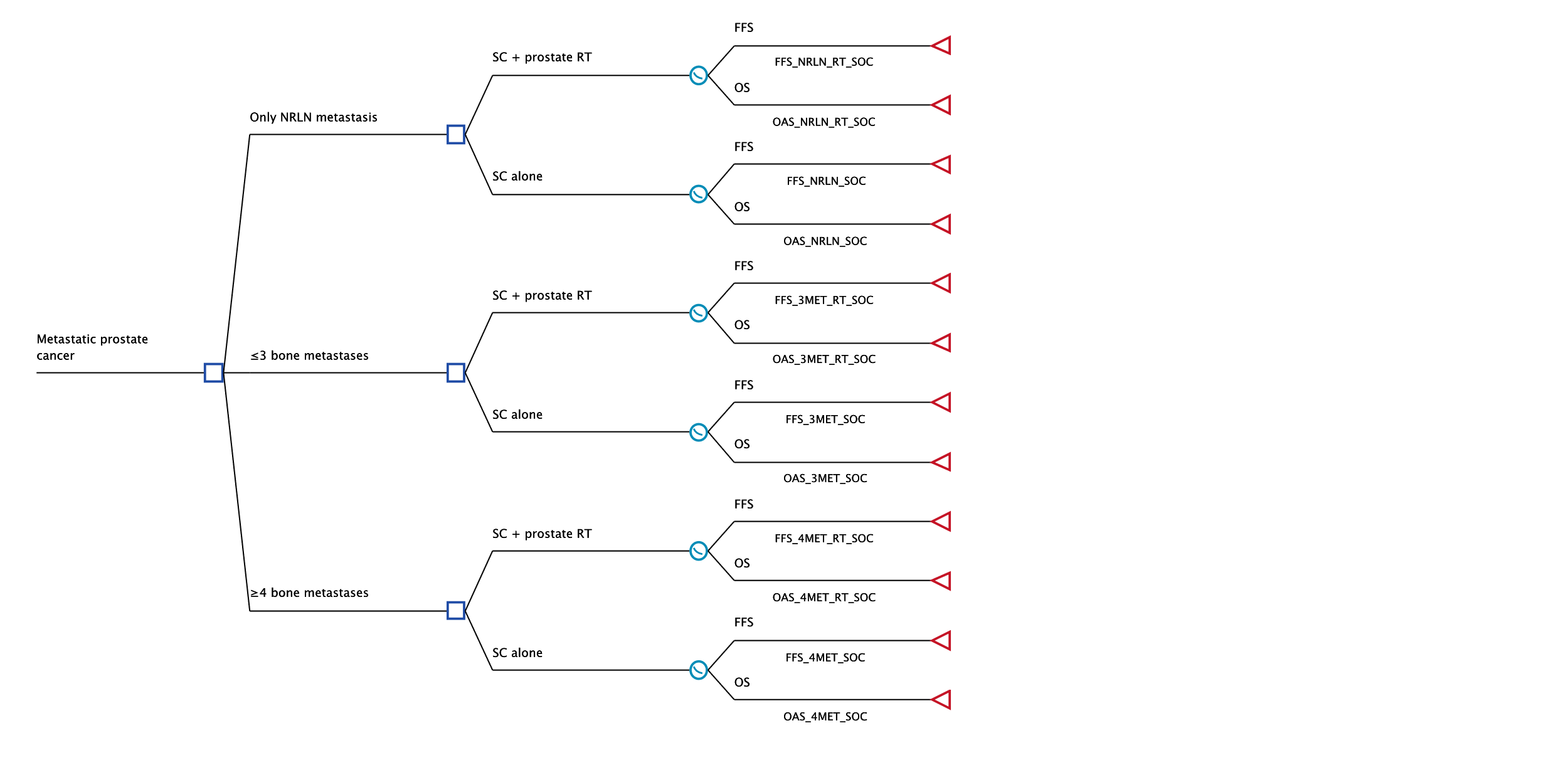


Model overview: a decision model for strategies SOC and SOC+RT for only NRLN metastases, ≤ 3 bone metastases and ≥ 4 metastases. For every outcome, a PSA analysis was carried out.

# Supplementary table S1 | Detailed cost calculation

## SOC FFS [1, 2]

| **type** | **used for patients [%]** | **drug** | **initial cost per drug** | **monthly cost per drug** | **initial cost** | **monthly cost** |
| --- | --- | --- | --- | --- | --- | --- |
| **LHRH agonists** | 100% |  |  |  |  | 619.53 USD |
|  |  | Goserelin |  | 494.28 USD |  |  |
|  |  | Leuprorelin |  | 744.79 USD |  |  |
| **Dual androgen blockage** |  |  |  |  |  |  |
|  | 27% | Enzalutamide |  | 3,432.27 USD |  | 938.15 USD |
|  | 27% | Apalutamide |  | 3,568.22 USD |  | 975.31 USD |
|  | 27% | Abiraterone |  | 1,856.14 USD |  | 507.34 USD |
| **Additional therapy** |  |  |  |  |  |  |
|  | 18% | Dozetaxel | 18,216.64 USD |  | 3.278,99 USD |  |
| **Total** |  |  |  |  | 3,278,99 USD | 3,040.34 USD |

## SOC post-progression [1, 2]

| **Type** | **used for patients [%]** | **drug** | **initial cost per drug** | **monthly cost per drug** | **initial cost** | **monthly cost** |
| --- | --- | --- | --- | --- | --- | --- |
| **LHRH agonists** | 100% |  |  |  |  | 619.53 USD |
|  |  | goserelin |  | 494.28 USD |  |  |
|  |  | leuprorelin |  | 744.79 USD |  |  |
| **Additional therapy (by percent)** | | | | | | |
|  | 60% | Enzalutamide |  | 3,432.27 USD |  | 2,059.36 USD |
|  | 21% | Abiraterone |  | 1,856.14 USD |  | 389.79 USD |
|  | 6% | Cabazitaxel | 60,132.67 USD |  | 3,607.96 USD |  |
|  | 33% | Docetaxel | 18,216.64 USD |  | 6,011.49 USD |  |
| **Total** |  |  |  |  | 9,619.45 USD | 3,068.68 USD |

## RT 20 fractions [1, 3, 4]

| **Steps** | **HCPCS Code** | **Short Description** | **Non-Facility Price [USD]** | **Units** | **Total price** |
| --- | --- | --- | --- | --- | --- |
| **Preparing for treatment** | | | | | |
| Clinical treatment planning | 77263 | Radiation therapy planning | 170.26 USD | 1 | 170.26 USD |
| Simulation | 77290 | Set radiation therapy field | 469.26 USD | 1 | 469.26 USD |
| **Medical Radiation Physics, Dosimetry, Treatment Devices, and Special Services** | | | | | |
| 3-dimensional radiotherapy plan | 77295 | 3-d radiotherapy plan | 482.76 USD | 1 | 482.76 USD |
| Basic dosimetry | 77300 | Radiation therapy dose plan | 66.10 USD | 1 | 66.10 USD |
| IMRT dose planning | 77301 | Radiotherapy dose plan imrt | 1,863.89 USD | 1 | 1,863.89 USD |
| MLC device for IMRT | 77338 | Design mlc device for imrt | 466.15 USD | 1 | 466.15 USD |
| Continuing medical physics consultation | 77336 | Radiation physics consult | 84.09 USD | 4 | 336.36 USD |
| **Radiation Treatment Delivery** | | | | | |
| IMRT treatment delivery (G-codes) | G6015 | Radiation tx delivery imrt | 375.13 USD | 20 | 7,502.60 USD |
| **Radiation Treatment Management** | | | | | |
| Radiation treatment management | 77427 | Radiation tx management | 192.76 USD | 4 | 771.04 USD |
| **Total Medicare Fee** | | | | | **12,128.42 USD** |

## RT 6 fractions [1, 3, 4]

| **Steps** | **HCPCS Code** | **Short Description** | **Non-Facility Price [USD]** | **Units** | **Total price** |
| --- | --- | --- | --- | --- | --- |
| **Preparing for treatment** | | | | | |
| Clinical treatment planning | 77263 | Radiation therapy planning | 170.26 USD | 1 | 170.26 USD |
| Simulation | 77290 | Set radiation therapy field | 469.26 USD | 1 | 469.26 USD |
| **Medical Radiation Physics, Dosimetry, Treatment Devices, and Special Services** | | | | | |
| 3-dimensional radiotherapy plan | 77295 | 3-d radiotherapy plan | 482.76 USD | 1 | 482.76 USD |
| Basic dosimetry | 77300 | Radiation therapy dose plan | 66.10 USD | 1 | 66.10 USD |
| IMRT dose planning | 77301 | Radiotherapy dose plan imrt | 1,863.89 USD | 1 | 1,863.89 USD |
| MLC device for IMRT | 77338 | Design mlc device for imrt | 466.15 USD | 1 | 466.15 USD |
| Continuing medical physics consultation | 77336 | Radiation physics consult | 84.09 USD | 6 | 504.54 USD |
| **Radiation Treatment Delivery** | | | | | |
| IMRT treatment delivery (G-codes) | G6015 | Radiation tx delivery imrt | 375.13 USD | 6 | 2,250.78 USD |
| **Radiation Treatment Management** | | | | | |
| Radiation treatment management | 77427 | Radiation tx management x5 | 192.76 USD | 6 | 1,156.56 USD |
| **Total Medicare Fee** |  |  |  |  | **7,430.30** **USD** |

Detailed cost calculations for SOC in failure-free state and after progression as well as RT in two different therapy regimes. For the analysis the more expensive regime for RT was utilized.

# Supplementary table S2 | Detailed utility calculations

|  | **Average utility value** | **Detailed** |
| --- | --- | --- |
| **SOC** | 0.82 | Cooperberg et al. [5] (0.92)  Hall et al. [6] (0.71) Sathianathen et al. [7] (0.83) |
| **SOC+RT** | 0.9 | Lester-Coll et al. [8] (0.9) |
| **Genitourinary (GU) and gastrointestinal (GI) late toxicities** | 0.993* | **GU** (1-0.145), prevalence 1.72 %  Cooperberg et al. [5] (1-0.15)  Sanyal et al. [9] (1-0.08)  Alyamani et al. [10] (1-0.25)  Lester-Coll et al. [8](1-0.10)  **GI** (1-0.188), prevalence 2.43 %  Cooperberg et al. [5] (1-0.2)  Sanyal et al. [9] (1-0.09)  Alyamani et al. [10] (1-0.25)  Lester-Coll et al. [8](1-0.21) |
| **Progress** | 0.6325 | Cooperberg et al. [5] (0.78)  Sanyal et al. [9] (0.60)  Lester-Coll et al. [8] (0.70)  Sathianathen et al. [7] (0.45) |

Detailed cost calculations for utilities in failure-free state, after progression and late toxicities. *Adjusted according to the declared late radiotherapy toxicity scores from Parker et al. (original STAMPEDE trial) Table 5 [11]. Disutilities were converted to utilities: Utilities = 1 – Disutilities.

# Supplementary table S4 | Monte Carlo simulations – Numeric results

|  | **NRLN metastases** | **≤ 3 metastases** |
| --- | --- | --- |
| **Preferred strategy** |  |  |
| - SOC+RT (20 fractions) | 85.72 % | 84.80 % |
| - SOC | 14.28 % | 15.20 % |
| **ICER < 100.000 USD/QALY for SOC+RT** | 96.72 % | 99.61 % |

Monte Carlo simulations. Percentage of preferred strategies and ICER-values < 100.000 USD/QUALY of SOC vs. SOC+RT are shown for the Monte Carlo simulations.

# Supplementary table S5 | Comparison with other CEAs

| **Cost** | **Period** | **Unit** | **Value** | **Notes** |
| --- | --- | --- | --- | --- |
| **Wang et al. [12] – input parameters** | | | | |
| Costs mCSPC | Yearly cost | 2020 USD | 43,760.08 | [2, 13] |
|  | Monthly cost² | 2020 USD | 3,646.67 |  |
| Costs mCRPC (docetaxel, apalutamide, enzalutamide) (≈ progressive disease) | Yearly cost | 2020 USD | 147,548.80 | [2, 13] |
|  | Monthly cost² | 2020 USD | 12,295.73 |  |
| Utility mCSPC |  |  | 0.8 | [14] |
| Utility mCRPC |  |  | 0.716 | [15] |
| **Lester-Coll et al. [8] – input parameters** | | | | |
| ADT plus PRT once | One time costs | USD | 16,860 | Institutional Medicare fees |
| ADT monthly | Monthly cost | USD | 63 | Pollard et al, 2017 [16] |
| Progression 1 | Monthly cost | USD | 5,738 | Pollard et al, 2017 [16] |
| Progression 2 | Monthly cost | USD | 17,365 | Pollard et al, 2017 [16] |
| Toxic effects associated with radiation therapy once | One time costs | USD | 2,208 |  |
| RT 20 fractions | One time costs | USD | 16,337.90 |  |
| RT 6 fractions | One time costs | USD | 9,658.58 |  |
| ADT plus PRT |  |  | 0.90 | [17] |
| PRT |  |  | 0.83 | [17] |
| **Clarke et al. [18] – base case results** | | | | |
| AAP+SOC M1 subgroup | Lifetime costs | GBP | 116,658 |  |
| AAP+SOC M1 subgroup | Lifetime costs | USD^1^ | 157,856 |  |
| SOC only M1 subgroup | Lifetime costs | GBP | 46,412 |  |
| SOC only M1 subgroup | Lifetime costs | USD^1^ | 62,802 |  |
| **Kramer et al. – input parameters** | | | | |
| SOC initial costs | One time costs | USD | 3,278.99 |  |
| SOC monthly costs | Monthly cost | USD | 3,040.34 |  |
| SOC yearly costs | Yearly cost | USD | 36,484.08 |  |
| RT 20 fractions | One time costs | USD | 12,128.42 |  |
| RT 6 fractions | One time costs | USD | 7,430.30 |  |
| Progression – SOC initial costs | Initial costs | USD | 9,619.45 |  |
| Progression – SOC monthly costs | Monthly costs | USD | 3,068.68 |  |
| Utility SOC |  |  | 0.82 |  |
| Utility SOC+RT |  |  | 0.90 |  |
| Utility late toxicities |  |  | 0.99 |  |
| Utility progress |  |  | 0.6325 |  |
| **Kramer et al. – base case results** | | | | |
| NRLN – SOC | Lifetime costs |  | 154,787.61 |  |
| NRLN – SOC+RT | Lifetime costs |  | 180,885.86 |  |
| ≤ 3 bone metastases – SOC | Lifetime costs |  | 173,899.48 |  |
| ≤ 3 bone metastases – SOC + RT | Lifetime costs |  | 327,389.10 |  |
| ≥ 4 bone metastases – SOC | Lifetime costs |  | 195,500.90 |  |
| ≥ 4 bone metastases – SOC + RT | Lifetime costs |  | 180,639.41 |  |
| Average – SOC |  |  | 174,729.33 |  |
| Average SOC+RT |  |  | 229,638.12 |  |

Comparison of costs with other studies as external validation. A detailed overview of assumed costs from different studies. Costs are shown in USD. AAP = abiraterone acetate plus prednisolone; ^1^ Exchange rate GBP to USD on 01.01.2022 (source: Bankenverband.de), ² Calculated by dividing yearly cost by 12 months.

# Supplementary table S6 | Comparison of base case analysis with Lester-Coll [19]

|  | **Treatment arm** | **Cost [USD]** | **QALYs** | **Increased cost [USD]** | **Increased QALYs** |
| --- | --- | --- | --- | --- | --- |
| **Kramer et al.** | | | | | |
| NRLN | SOC | 154,788 | 2.89 |  |  |
|  | SOC+RT | 180,886 (176,188) | 3.81 | 26,098 (21,400) | 0.92 |
| ≤ 3 bone metastases | SOC | 173,899 | 3.24 |  |  |
|  | SOC+RT | 327,389 (322,691) | 7.07 | 153,490 (148,792) | 3.83 |
| ≥ 4 bone metastases | SOC | 195,501 | 3.44 | 14,861 (19,559) | 0.03 |
|  | SOC+RT | 180,639 (175,941) | 3.41 |  |  |
| **Lester-Coll et al. [19]** | | | | | |
| 37 month follow up | SOC | 114,223 | 1.62 |  |  |
|  | SOC + RT | 94,751 | 1.78 | -19,472 | 0.16 |
| lifetime costs | SOC | 328,971 | 2.22 |  |  |
|  | SOC + RT | 298,741 | 3.03 | -30,229 | 0.81 |

Comparison of base case analysis with Lester-Coll et al. **[19].**  Values in brackets “()” are calculated for the more inexpensive SOC+RT with a 6 fractions RT schedule. At time of analysis, the description of the therapy regimes in the table seemed to be inconclusive to the text, where the comparison between SOC and SOC+RT was described. With information from the text values were reconstructed and completed using logical conclusions and table 2 of their base case results. If a second erratum is to be published by Lester-Coll et al., this might be identified directly.

# Supplementary figure S2 | Parametric distributions

|  | **Values** | **Distribution** | **Fittings** |
| --- | --- | --- | --- |
| ***SOC*** |  |  |  |
| FFS only NRLN metastases | mean = 3.047  sd = 1.267 | LogNormal | 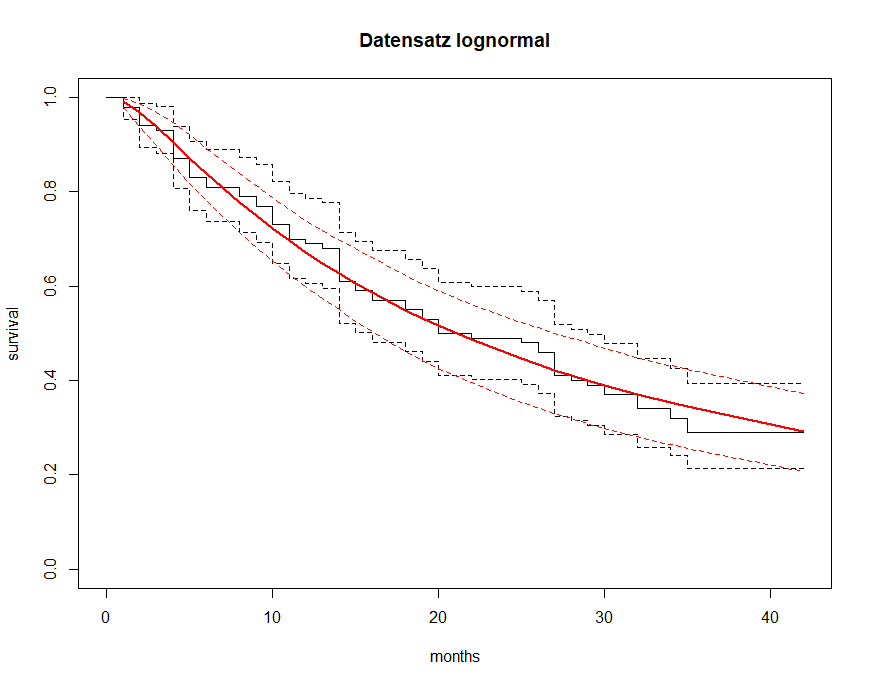 |
| OS only NRLN metastases | shape = 0.068548  rate = 0.001768 | Gompertz | 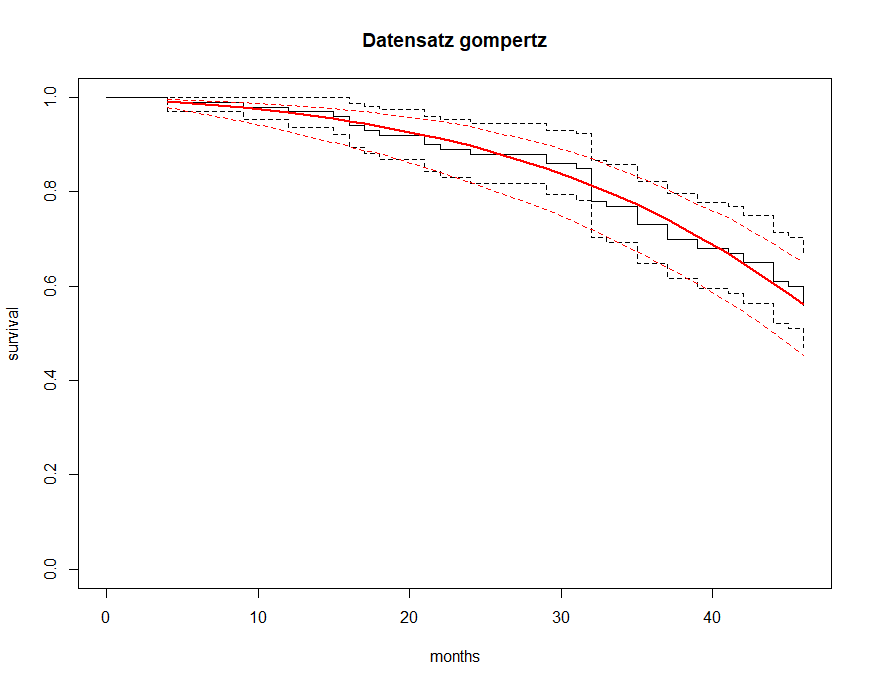 |
| FFS ≤ 3 metastases | shape = 0.00417  rate = 0.02929 | Gompertz | 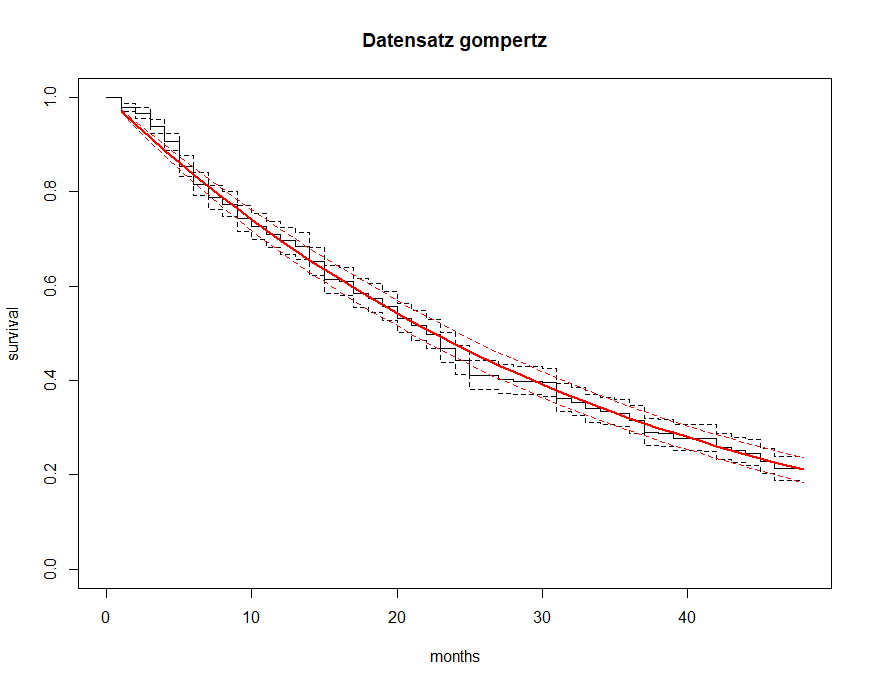 |
| OS ≤ 3 metastases | shape = 0.043778  rate = 0.003057 | Gompertz | 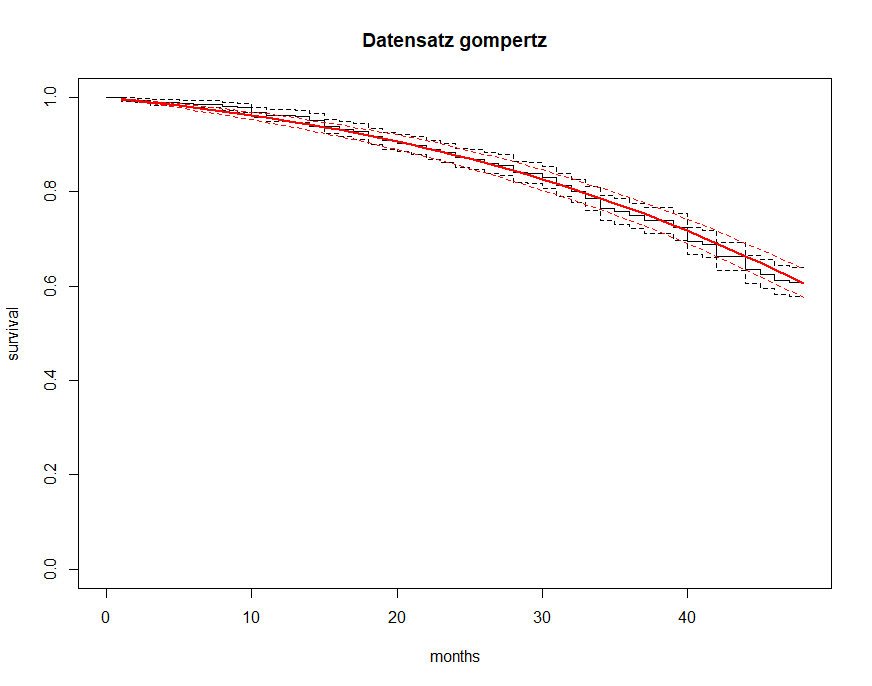 |

|  | **Values** | **Distribution** | **Fittings** |
| --- | --- | --- | --- |
| FFS ≥ 4 metastases | mean = 2.3222  sd = 1.1322 | LogNormal | 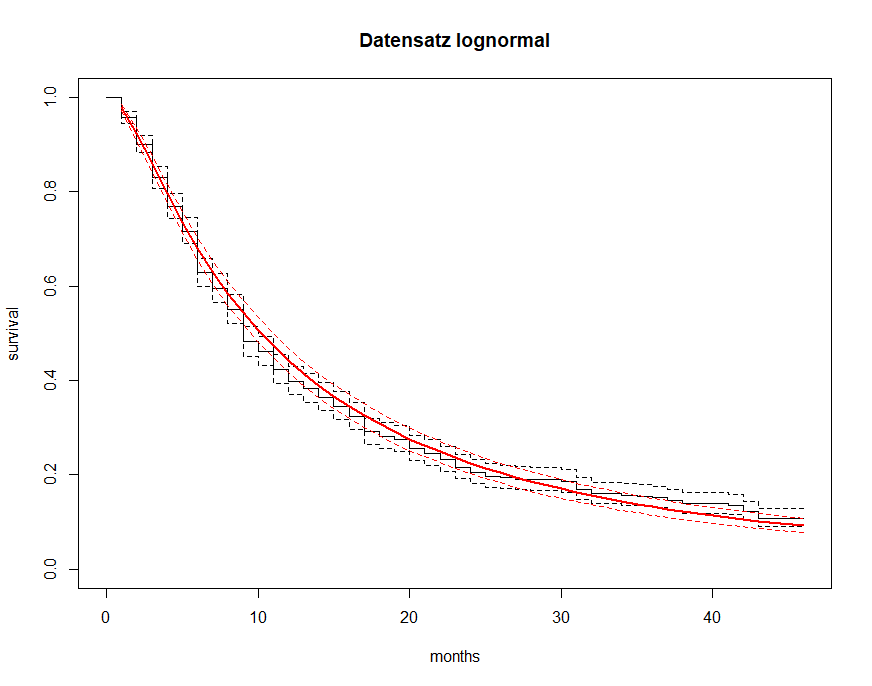 |
| OS ≥ 4 metastases | mean = 3.7020  sd = 0.8840 | LogNormal | 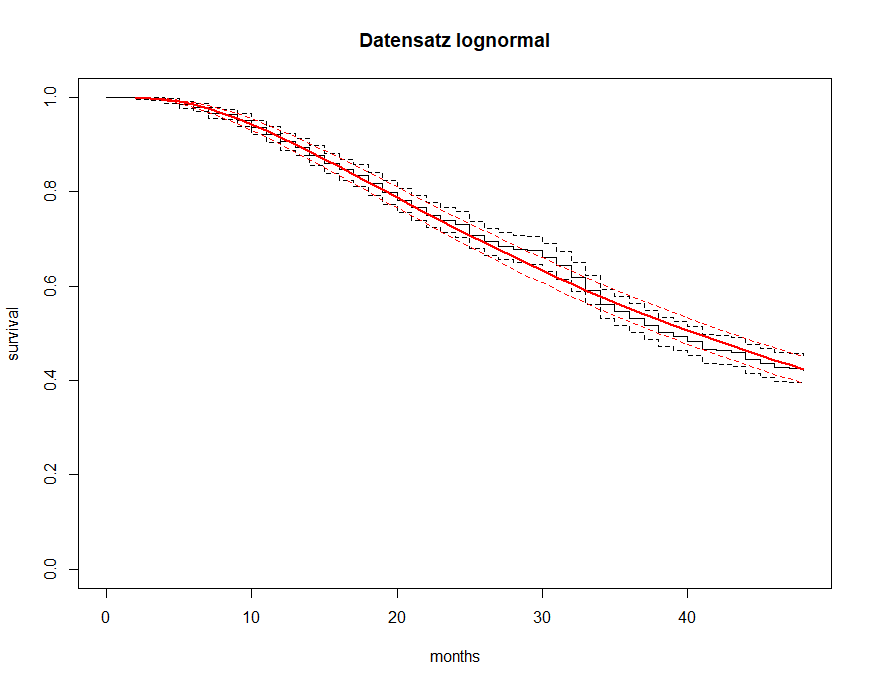 |
|  |  |  |  |
| ***SOC+RT*** |  |  |  |
| FFS only NRLN metastases | mean = 3.575  sd = 1.135 | LogNormal | 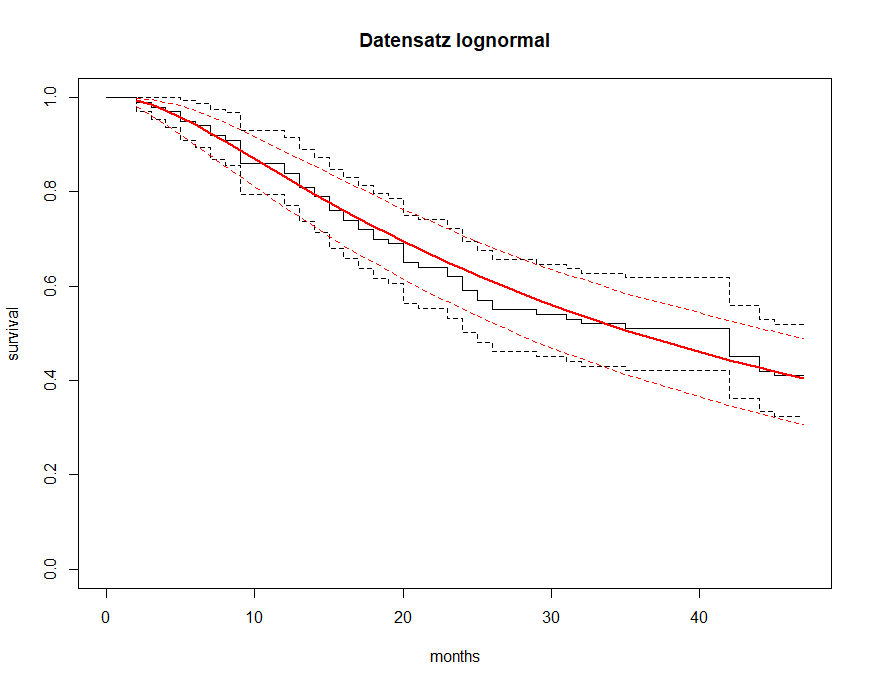 |
| OS only NRLN metastases | shape = 0.070110  rate = 0.001177 | Gompertz | 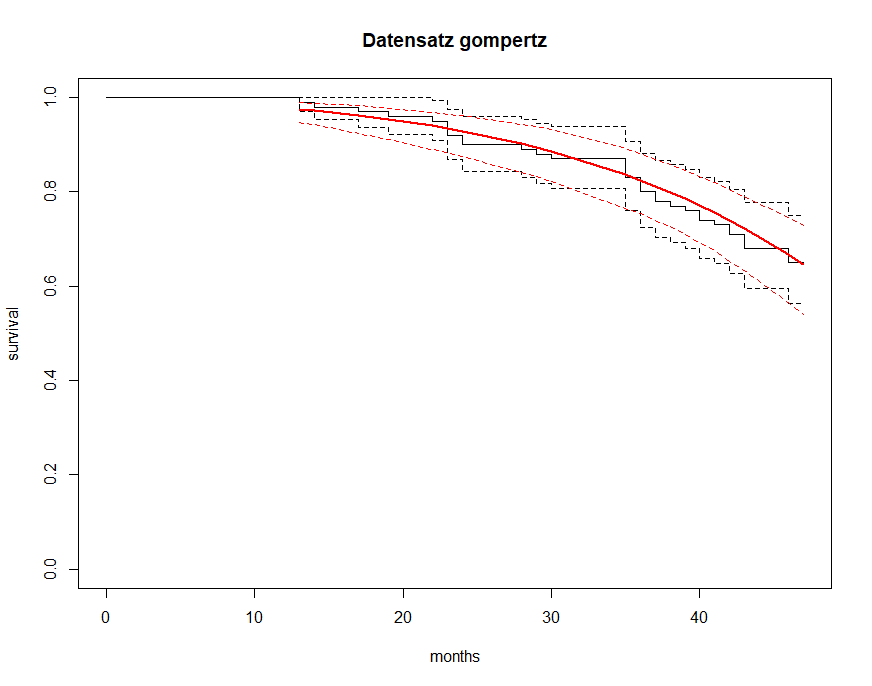 |

|  | **Values** | **Distribution** | **Fittings** |
| --- | --- | --- | --- |
| FS ≤ 3 metastases | mean = 3.6178  sd = 1.2795 | LogNormal | 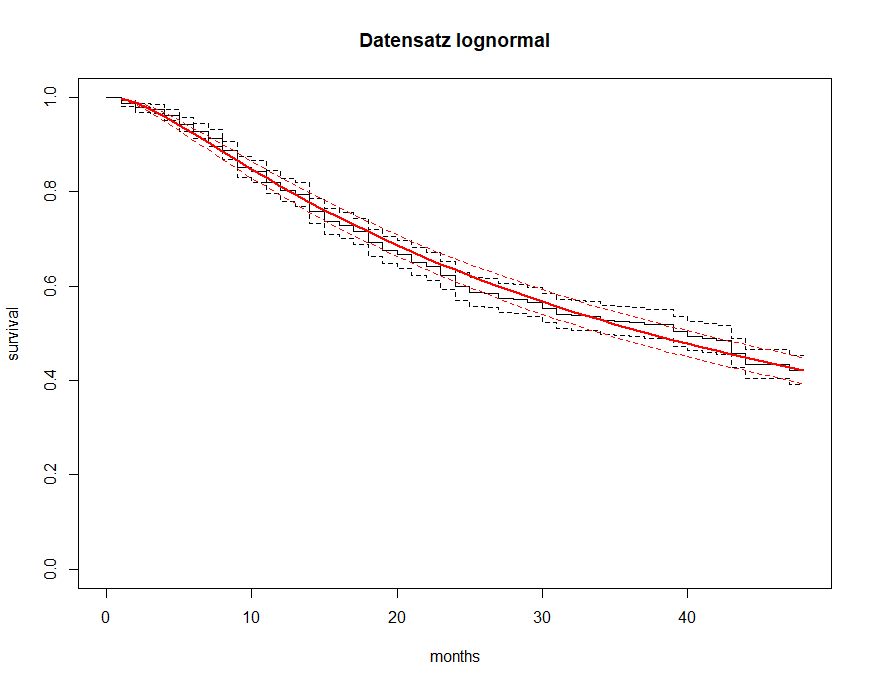 |
| OS ≤ 3 metastases | mean = 4.3119 sd = 0.7626 | LogNormal | 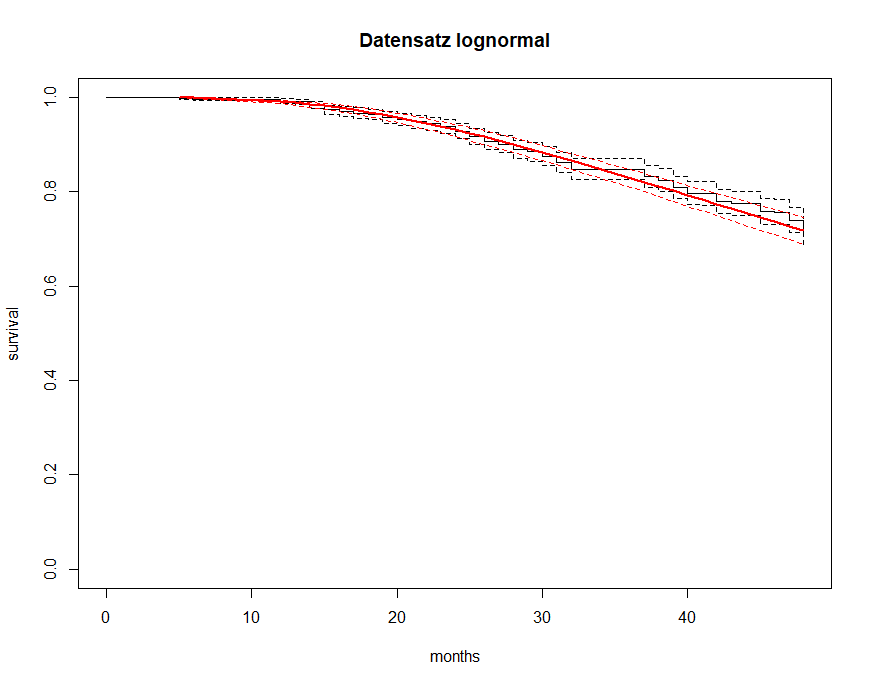 |
| FFS ≥ 4 metastases | mean = 2.4377  sd = 1.1448 | LogNormal | 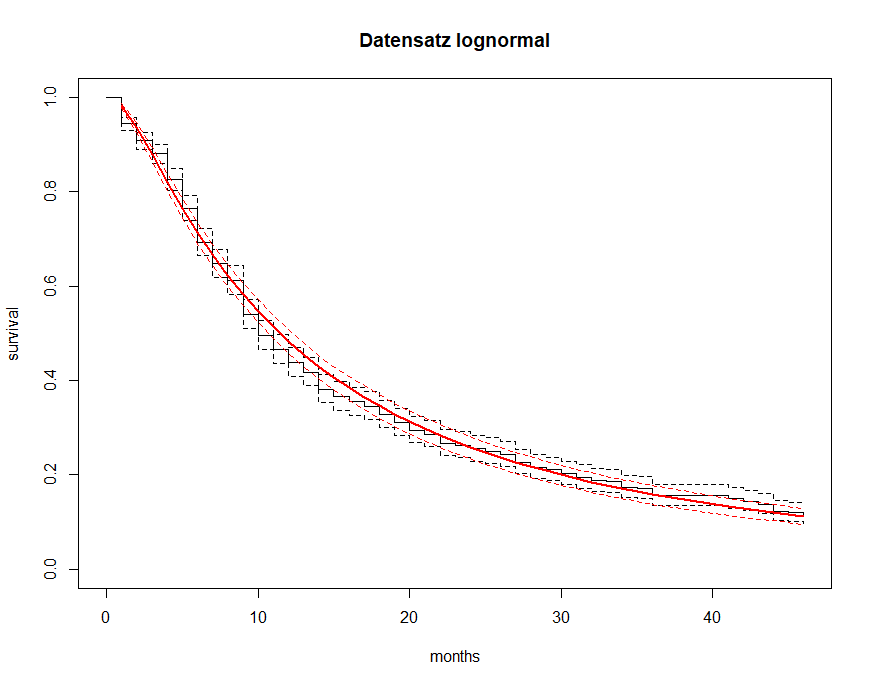 |
| OS ≥ 4 metastases | mean = 3.6211  sd = 0.7869 | LogNormal | 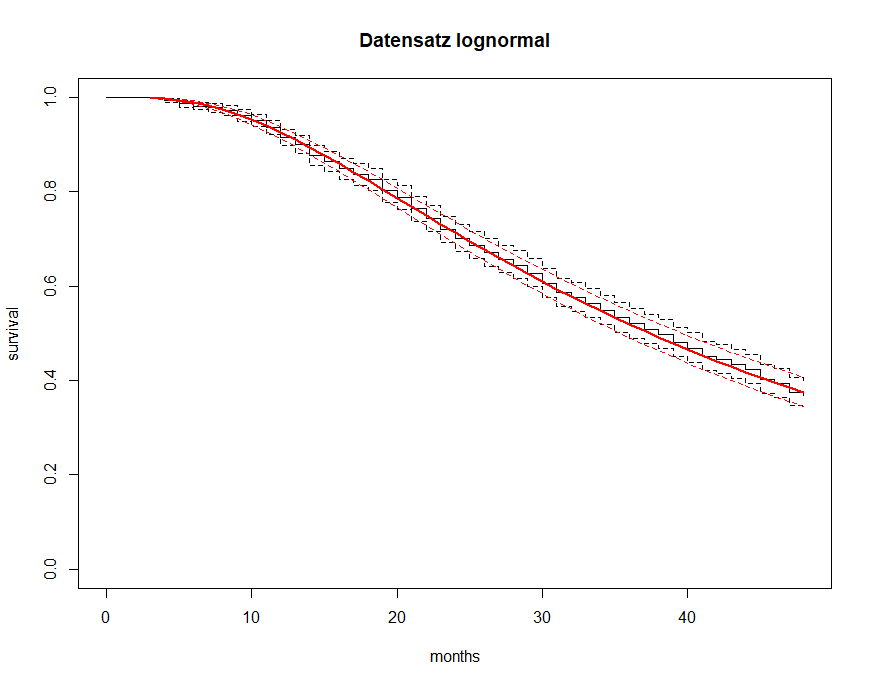 |

Parametric distributions on the extracted survival curves of Ali et al. [20]. The parameters, the distributions, and the plot of the distribution on the extracted survival curves are displayed.

# Supplementary text

## Introduction

### Therapies

Whereas local tumors are treated primarily with local therapies [21], the current standard of care (SOC) treatment for advanced castration-sensitive prostate cancer (CSPC) is androgen-deprivation therapy (ADT) in combination with a second antiandrogenic active treatment [21, 22]. Furthermore, these malignancies tend to develop a resistance against ADT and progress to metastatic castrate-resistant prostate cancers (mCRPC) [23, 24], resulting in a daunting prognosis with a median survival between 10 and 18 months after initial treatment with docetaxel [25].

## Materials and Methods

### Cost-Effectiveness Analysis

Effectiveness is being quantified as quality-adjusted life year (QALYs). One QALY equals to living one year in perfect health. Total amount of QALYs per treatment arm were calculated by multiplying time spent in stable disease and progressive disease states and assigned utility weights.

$QALY = time\times utility$ [26]

The ICER represents a measure for cost-effectivity. It expresses the cost necessary for gaining one QALY.

$ICER= \frac{incremental cost}{incremental benefit}=\frac{cost of strategy A - cost of strategy B}{benefit of strategy A- benefit of strategy B}$ [27]

The net monetary benefit (NMB) combines cost and effectiveness:

$NMB= (effectivity\times willingness to pay) - cost$ [26]

If the ICER is below the willingness to pay threshold of one QALY the NMB is positive. This indicates that the intervention is cost-effective compared to the alternative treatment [28]. The willingness-to-pay threshold was set to 100.000 USD per QALY, according to literature [29]. This threshold value classifies medical services in terms of reimbursability [27].

## Discussion

### Clinical context of additional RT in patients with metastatic prostate cancer

A contemporary analysis by Rusthoven et al. [30] showed that men with metastatic prostate cancer receiving additional prostate RT have shown a better survival prognosis than men treated with ADT alone. The study was based on the National Cancer Database (NCDB) to evaluate the overall survival, while being consistent to the findings of STAMPEDE [11].

Morgan et al. [31] investigated the long-term outcomes of prostate radiotherapy for newly diagnosed metastatic prostate cancer, based on data from their study center. An improved overall survival with a clinically significant magnitude of the OS benefit was revealed in patients who received additional prostate RT.

Besides applications in the US, Dai et al. 2022 [32] in Shanghai, China, performed a phase II randomized controlled trial including patients with newly diagnosed oligometastatic prostate cancer. Significantly higher rates of progression-free survival and OS for patients who received a radical local therapy (radical prostatectomy or prostate radiation therapy) were found compared to patients who received ADT alone.

In a long-term analysis of the STAMPEDE trial the efficiency of the RT treatment could be further endorsed [33], validating the findings of this analysis.

# References

1. Medicare & Medicaid Spending by Drug. [cited 2022 July 10]; Available from: <https://data.cms.gov/summary-statistics-on-use-and-payments/medicare-medicaid-spending-by-drug>

2. Physician fee schedule search. Center for Medicare and Medicaid Services. [cited 2021 January 4]; Available from: <https://www.cms.gov/apps/physician-fee-schedule/search/search-criteria.aspx>.

3. NCCN Clinical Practice Guidelines in Oncology (NCCN Guidelines®) Version 4.2022. [cited 2022 November 18]; Available from: <https://www.nccn.org/professionals/physician_gls/pdf/prostate.pdf>.

4. RADIATION ONCOLOGY CPT® and HCPCS CODES BY PROCESS OF CARE. [cited 2022 July 10]; Available from: <https://www.astro.org/uploadedFiles/_MAIN_SITE/Daily_Practice/Reimbursement/Practice_Management_Resources/Content_Pieces/CPTCodeChart.pdf>

5. Cooperberg, M.R., et al., Primary treatments for clinically localised prostate cancer: a comprehensive lifetime cost-utility analysis. BJU Int, 2013. 111(3): p. 437-50.

6. Hall, F., et al., Estimating utilities/disutilities for high-risk metastatic hormone-sensitive prostate cancer (mHSPC) and treatment-related adverse events. Qual Life Res, 2019. 28(5): p. 1191-1199.

7. Sathianathen, N.J., et al., A Cost-effectiveness Analysis of Systemic Therapy for Metastatic Hormone-sensitive Prostate Cancer. Eur Urol Oncol, 2019. 2(6): p. 649-655.

8. Lester-Coll, N.H., et al., Supplementary Online Content of Cost-effectiveness of Prostate Radiation Therapy for Men With Newly Diagnosed Low-Burden Metastatic Prostate Cancer. JAMA Netw Open, 2021. 4(1): p. e2033787.

9. Sanyal, C., et al., Management of localized and advanced prostate cancer in Canada: A lifetime cost and quality-adjusted life-year analysis. Cancer, 2016. 122(7): p. 1085-96.

10. Alyamani, N., et al., Cost-Utility Analysis of Radiation Treatment Modalities for Intermediate-Risk Prostate Cancer. Curr Oncol, 2021. 28(4): p. 2385-2398.

11. Parker, C.C., et al., Radiotherapy to the primary tumour for newly diagnosed, metastatic prostate cancer (STAMPEDE): a randomised controlled phase 3 trial (article and/or supplementary material). Lancet, 2018. 392(10162): p. 2353-2366.

12. Wang, L., et al., Cost-Effectiveness of Systemic Treatments for Metastatic Castration-Sensitive Prostate Cancer: An Economic Evaluation Based on Network Meta-Analysis. Value Health, 2022. 25(5): p. 796-802.

13. Pharmaceutical Prices. U.S. Department of Veterans’ Affairs. [cited 2020 December 31]; Available from: <https://www.va.gov/opal/nac/fss/pharmPrices.asp>.

14. Chi, K.N., et al., Patient-reported outcomes following abiraterone acetate plus prednisone added to androgen deprivation therapy in patients with newly diagnosed metastatic castration-naive prostate cancer (LATITUDE): an international, randomised phase 3 trial. Lancet Oncol, 2018. 19(2): p. 194-206.

15. Lloyd, A.J., et al., Health-Related Quality of Life and Health Utilities in Metastatic Castrate-Resistant Prostate Cancer: A Survey Capturing Experiences from a Diverse Sample of UK Patients. Value Health, 2015. 18(8): p. 1152-7.

16. Pollard, M.E., et al., Cost-effectiveness analysis of treatments for metastatic castration resistant prostate cancer. Asian J Urol, 2017. 4(1): p. 37-43.

17. Stewart, S.T., et al., Utilities for prostate cancer health states in men aged 60 and older. Med Care, 2005. 43(4): p. 347-55.

18. Clarke, C.S., et al., Cost-utility analysis of adding abiraterone acetate plus prednisone/prednisolone to long-term hormone therapy in newly diagnosed advanced prostate cancer in England: Lifetime decision model based on STAMPEDE trial data. PLoS One, 2022. 17(6): p. e0269192.

19. Lester-Coll, N.H., et al., Cost-effectiveness of Prostate Radiation Therapy for Men With Newly Diagnosed Low-Burden Metastatic Prostate Cancer (article and/or supplementary material). JAMA Netw Open, 2021. 4(1): p. e2033787.

20. Ali, A., et al., Association of Bone Metastatic Burden With Survival Benefit From Prostate Radiotherapy in Patients With Newly Diagnosed Metastatic Prostate Cancer: A Secondary Analysis of a Randomized Clinical Trial. JAMA Oncol, 2021. 7(4): p. 555-563.

21. Mottet, N., et al., EAU-EANM-ESTRO-ESUR-SIOG Guidelines on Prostate Cancer-2020 Update. Part 1: Screening, Diagnosis, and Local Treatment with Curative Intent. Eur Urol, 2021. 79(2): p. 243-262.

22. Virgo, K.S., R.B. Rumble, and J. Talcott, Initial Management of Noncastrate Advanced, Recurrent, or Metastatic Prostate Cancer: ASCO Guideline Update. J Clin Oncol, 2023: p. Jco2300155.

23. Sartor, O. and J.S. de Bono, Metastatic Prostate Cancer. N Engl J Med, 2018. 378(7): p. 645-657.

24. Sweeney, C.J., et al., Chemohormonal Therapy in Metastatic Hormone-Sensitive Prostate Cancer. N Engl J Med, 2015. 373(8): p. 737-46.

25. Teo, M.Y., D.E. Rathkopf, and P. Kantoff, Treatment of Advanced Prostate Cancer. Annu Rev Med, 2019. 70: p. 479-499.

26. Mehrens, D., et al., Cost-Effectiveness Analysis of Local Treatment in Oligometastatic Disease. Front Oncol, 2021. 11: p. 667993.

27. Froelich, M.F., et al., Cost-effectiveness analysis in radiology: methods, results and implications. Rofo, 2022. 194(1): p. 29-38.

28. Net Monetary Benefit [online]. 2016 [cited 2023 July 25]; Available from: <https://yhec.co.uk/glossary/net-monetary-benefit/>.

29. Cameron, D., J. Ubels, and F. Norström, On what basis are medical cost-effectiveness thresholds set? Clashing opinions and an absence of data: a systematic review. Glob Health Action, 2018. 11(1): p. 1447828.

30. Rusthoven, C.G., et al., Improved Survival With Prostate Radiation in Addition to Androgen Deprivation Therapy for Men With Newly Diagnosed Metastatic Prostate Cancer. J Clin Oncol, 2016. 34(24): p. 2835-42.

31. Morgan, S.C., et al., Long-term outcomes of prostate radiotherapy for newly-diagnosed metastatic prostate cancer. Prostate Cancer Prostatic Dis, 2021. 24(4): p. 1041-1047.

32. Dai, B., et al., Combination of Androgen Deprivation Therapy with Radical Local Therapy Versus Androgen Deprivation Therapy Alone for Newly Diagnosed Oligometastatic Prostate Cancer: A Phase II Randomized Controlled Trial. Eur Urol Oncol, 2022. 5(5): p. 519-525.

33. Parker, C.C., et al., Radiotherapy to the prostate for men with metastatic prostate cancer in the UK and Switzerland: Long-term results from the STAMPEDE randomised controlled trial. PLoS Med, 2022. 19(6): p. e1003998.
